# Is the Proportion of Carbohydrate Intake Associated with the Incidence of Diabetes Complications?—An Analysis of the Japan Diabetes Complications Study

**DOI:** 10.3390/nu9020113

**Published:** 2017-02-06

**Authors:** Chika Horikawa, Yukio Yoshimura, Chiemi Kamada, Shiro Tanaka, Sachiko Tanaka, Satoshi Matsunaga, Osamu Hanyu, Atsushi Araki, Hideki Ito, Akira Tanaka, Yasuo Ohashi, Yasuo Akanuma, Hirohito Sone

**Affiliations:** 1 Department of Health and Nutrition, University of Niigata Prefecture Faculty of Human Life Studies, Niigata 950-8680, Japan; horikawa@unii.ac.jp; 2 Department of Hematology, Endocrinology and Metabolism, Niigata University Faculty of Medicine, Niigata 951-8510, Japan; smhm@med.niigata-u.ac.jp (S.M.); hanyuo@med.niigata-u.ac.jp (O.H.); 3 Training Department of Administrative Dietitians, Shikoku University, Tokushima, Tokushima 771-1151, Japan; yyoshimura@shikoku-u.ac.jp (Y.Y.); c-kamada@shikoku-u.ac.jp (C.K.); 4 Department of Pharmacoepidemiology, Graduate School of Medicine and Public Health, Kyoto University, Kyoto 606-8501, Japan; tanaka.shiro.8n@kyoto-u.ac.jp; 5 Department of Public Health, Shiga University of Medical Science, Sihga 520-2192, Japan; sachikot@belle.shiga-med.ac.jp; 6 Department of Endocrinology and Metabolism, Tokyo Metropolitan Geriatric Hospital, Tokyo 173-0015, Japan; aaraki@tmghig.jp (A.A.) ; hideki_ito@tmghig.jp (H.I.); 7 Nutrition Clinic, Kagawa Nutrition University, Tokyo 170-8481, Japan; clinic@eiyo.ac.jp; 8 Department of Integrated Science and Engineering of Sustainable Society, Chuo University, Tokyo 113-0033, Japan; ohashi@epistat.m.u-tokyo.ac.jp; 9 The Institute for Adult Diseases, Asahi Life Foundation, Tokyo 103-0002, Japan; y-akanuma@asahi-life.or.jp

**Keywords:** type 2 diabetes, carbohydrate intake, diabetes complications

## Abstract

The appropriate proportions of macronutritional intake have been controversial in medical nutritional therapy for diabetes, and evidence of the effects of carbohydrate consumption on diabetes complications in prospective settings is sparse. We investigated the relationships between proportions of carbohydrate intake as the % of total energy and diabetes complications in a nationwide cohort of Japanese patients with type 2 diabetes aged 40–70 years with hemoglobin A1c ≥6.5%. The analysis was of 1516 responders to a baseline dietary survey assessed by the Food Frequency Questionnaire based on food groups. Primary outcomes were times to overt nephropathy, diabetic retinopathy, and cardiovascular disease (CVD) after 8 years. Hazard ratios (HRs) for proportions of carbohydrate intake were estimated by Cox regression adjusted for confounders. High carbohydrate intake was significantly related to higher intakes of grain, fruits, and sweets/snacks and lower intakes of soybean and soy products, vegetables, seaweed, meat and processed meat, fish and processed fish, eggs, milk and dairy products, oil, and alcoholic beverages. During the eight-year follow-up, there were 81, 275, and 129 events of overt nephropathy, diabetic retinopathy, and CVD, respectively. After adjustment for confounders, HRs for complications in patients with carbohydrate intake in the second or third tertiles (51.0%–56.4% and ≥56.5%, respectively) compared with carbohydrate intake in the first tertile (<50.9%, referent) were analyzed. No significant associations were shown in the second and third tertiles relative to first tertile (overt nephropathy: 1.05 (95% Confidence Interval, 0.54–2.06) and 0.98 (0.40–2.44); diabetic retinopathy: 1.30 (0.90–1.88) and 1.30 (0.78–2.15); and CVD: 0.95 (0.55–1.63) and 1.37 (0.69–2.72)). By exploring potentially nonlinear relationships, trends for the incidence of diabetes complications according to proportions of carbohydrate intake were not clearly shown. Findings suggested that proportions of carbohydrate intake were not associated with the incidence of diabetes complications among type 2 diabetes patients in Japan.

## 1. Introduction

The optimal proportion of dietary carbohydrate among macronutrients for patients with diabetes has been controversial with regard to medical nutritional therapy. A previous meta-analysis of short-term randomized controlled trials of patients with diabetes showed favorable effects of low-carbohydrate diets (median carbohydrate of total energy: 40%) on short-term clinical parameters for diabetes such as reductions in fasting insulin and triglycerides and increases in high-density lipoprotein cholesterol [1]. Another meta-analysis of population-based longitudinal studies of participants without diabetes suggested that low-carbohydrate diets were associated with a significantly higher risk of all-cause mortality [2]. Moreover to date, there have been no large scale studies examining the relationship between carbohydrate intake and diabetes complications in prospective settings. Therefore, epidemiological evidence of long-term effects of carbohydrate consumption in diabetes care is still sparse.

A position statement by the American Diabetes Association in 2016 [3] did not conclude that either high or low carbohydrate diets were preferable for prevention of diabetes complications because there has been no agreement on a single ideal dietary distribution of calories among macronutrients for patients with diabetes. The European Association for the Study of Diabetes and the Japan Diabetes Society (JDS) recommended ranges of carbohydrate intake from 45% to 60% and from 50% to 60%, respectively, although both organizations cited that more research was needed [4,5].

Establishing the appropriate dietary macronutrient composition, including carbohydrate intake, is among the most important points for understanding what and how much food should be consumed for a healthy life [6,7]. Consideration of the optimal proportion of carbohydrate intake by patients with diabetes using a longitudinal research design is urgently needed. Therefore, we investigated the association between proportions of carbohydrate intake and the incidence of diabetes complications including diabetic retinopathy, overt nephropathy, and cardiovascular disease (CVD) in patients with type 2 diabetes in the large nationwide multicenter cohort of the Japan Diabetes Complications Study (JDCS).

## 2. Materials and Methods

### 2.1. Study Cohort

The present analysis was conducted as part of the JDCS, a multicenter prospective study on the incidence of and risk factors for macro- and microvascular complications among Japanese patients with type 2 diabetes from outpatient clinics in 59 university and general hospitals in Japan. The primary results [8] of the JDCS were described elsewhere. Subjects considered eligible for inclusion were previously diagnosed individuals with type 2 diabetes aged 40–70 years whose hemoglobin A1c (HbA1c) levels were ≥6.5% and who were diagnosed by fasting blood glucose or the 75 g oral glucose tolerance test according to values established by the JDS. Assays for HbA1c were standardized by the Laboratory Test Committee of the JDS using values established by the JDS [5]; the National Glycohemoglobin Standardization Program (NSGP) value for HbA1c [9] would be calculated as follows: 0.25 + 1.02 × JDS value [10].

From January 1995 to March 1996, 2205 patients were initially registered in the JDCS. Of the 2033 patients who met the eligibility criteria described above, 1516 patients responded to a baseline dietary survey. There were no notable differences in baseline characteristics between responders and non-responders [11].

The original primary endpoints of the JDCS were micro- and macrovascular complications. The JDCS analyzed specified populations according to primary outcomes: CVD-incident group, overt nephropathy-incident group, and retinopathy-incident group. The CVD-incident group consisted of 1353 patients after excluding patients with impaired glucose tolerance, a history of angina pectoris, myocardial infarction, stroke, peripheral artery disease, familial hypercholesterolemia, type III hyperlipidemia (diagnosed by broad beta band on electrophoresis), or nephrotic syndrome (urine protein >3.5 g/day and serum total protein <6.0 mg/dL or serum creatinine levels >1.3 mg/dL (120 µmol/L) at baseline. The overt nephropathy-incident group consisted of 1275 patients after excluding those with impaired glucose tolerance, a history of non-diabetic nephropathy, nephrotic syndrome, serum creatinine levels >120 μmol/L, or mean values of two spot urine examinations for an albumin excretion rate of <150 mg/g creatinine. The retinopathy-incident group consisted of 936 patients after excluding those with impaired glucose tolerance, a history of retinopathy, or a major ocular disease (e.g., glaucoma, dense cataract, or history of cataract surgery). We analyzed follow-up data collected until March 2003.

The protocol for the study, which is in accordance with the Declaration of Helsinki and the Ethical Guidelines for Clinical/Epidemiological Studies of the Japanese Ministry of Health Labor and Welfare, received ethical approval from the institutional review boards of all of the participating institutes. Written informed consent was obtained from all enrolled patients.

### 2.2. Outcome Measures

CVD endpoints included the incidence of definite coronary heart disease (angina pectoris or myocardial infarction) or stroke. The diagnosis of angina pectoris and myocardial infarction was according to criteria defined by the WHO/MONICA (Multinational Monitoring of Trends and Determinants in Cardiovascular Disease) project, and the diagnosis of stroke was according to guidelines defined by the Ministry of Health, Labor and Welfare of Japan [12]. Adjudication of CVD endpoints was made by a central committee comprised of experts in each complication based on additional data such as a detailed history, sequential changes in ECG and serum cardiac biomarkers, and results of coronary angiography or brain imaging. The nephropathy endpoint was defined as the development of overt nephropathy (spot urinary albumin excretion >300 mg/g creatinine in two consecutive samples). Diabetic retinopathy was evaluated by qualified ophthalmologists at each institute using the following classification designed for this research: Stage 0, no retinopathy; Stage 1, hemorrhage and hard exudates; Stage 2, soft exudates; Stage 3, intraretinal microvascular abnormalities and venous changes including beading, loop, and duplication; and Stage 4, new vessels, vitreous hemorrhage, fibrous proliferation, and retinal detachment. The retinopathy endpoints were (i) development of retinopathy (from Stage 0 to any other stage confirmed in two continuous years) and (ii) progression from Stage 1 to Stage 3 or 4.

### 2.3. Dietary Assessment

Nutritional and food intakes were assessed by the Food Frequency Questionnaire based on food groups (FFQg) at baseline. In brief, the FFQg elicited information on the average intake per week of 29 food groups and 10 kinds of cookery in commonly used units or portion sizes. After participants completed the questionnaire, a dietitian reviewed the completed questionnaire with the participant. The FFQg was externally validated by comparison with dietary records for 7 continuous days of 66 participants aged 19–60 years [13]. The ratios of the estimates obtained by the FFQg against those by the dietary records ranged from 72% to 121% (average 104%). The correlation coefficient between the FFQg and dietary records for carbohydrate intake was 0.49. We used standardized software for population-based surveys and nutrition counseling in Tokushima, Japan (EIYO-KUN v.4.5, manufactured at the site of the Shikoku University Nutrition Database) based on Standard Tables of Food Composition in Japan [14] edited by the Japanese Ministry of Education, Culture, Sports, Science, and Technology to calculate nutrient and food intakes.

### 2.4. Statistical Analysis

Patients’ characteristics were described as mean ± standard deviation (SD), median ± interquartile range, or percentage. The first occurrence of overt nephropathy, diabetic retinopathy, or CVD was analyzed with time-to-event methods. Univariate and multivariate Cox regression analyses were used to estimate the adjusted hazard ratios (HR) and 95% Confidence Interval (CI) for each outcome after an 8-year follow-up in relation to the proportion of carbohydrate intake at baseline by conducting a tertile analysis and assigning the lowest tertile of carbohydrate intake as the referent. Multivariate adjusted analyses were conducted with adjustment for age, sex, body mass index (BMI), HbA1c, diabetes duration, systolic blood pressure, LDL-cholesterol, HDL-cholesterol, triglycerides, treatment with insulin, treatment by antihypertensive agents, treatment by lipid-lowering agents, current smoker, alcohol intake, energy intake, and physical activity. In addition to the multivariate adjustment, we made further adjustments for intakes of saturated fatty acids, monounsaturated fatty acids, polyunsaturated fatty acids, omega 6 fatty acids, omega 3 fatty acids, cholesterol, dietary fiber, and sodium. The distribution of nutritional intake was inspected and was not found to be irregular or physiologically unlikely, with the minimum and maximum of total energy intake being 638.8 kcal and 4056.8 kcal, respectively. All *p*-values are two-sided, and the significance level is 0.05. Potential nonlinear relationships between the proportion of carbohydrate intake and diabetes complications were explored by a spline function, a smooth curve for the incidence rate of diabetes complications depending on the proportion of carbohydrate intake. The spline function and 95% CI were estimated by energy-adjusted generalized additive models, and the degree of freedom was determined by generalized cross validation. All statistical analyses and data management were conducted at a central data center using SAS ver. 9.2 (SAS Institute Inc., Cary, NC, USA).

## 3. Results

Baseline clinical characteristics of the 1516 JDCS patients were grouped according to tertiles of carbohydrate intake as a percentage of total energy intake (Table 1) are shown. The age of participants and percentages of women tended to increase with increases in the proportion of carbohydrate intake (trend *p* < 0.01 and < 0.01, respectively). However, there were no significant trends in fasting blood glucose, % with diabetic retinopathy, BMI, systolic blood pressure, LDL-cholesterol, triglyceride levels, urine ACR, eGFR, prevalence of current smokers, and physical activity. There were significant differences in HbA1c and waist circumference from first to the third tertile of carbohydrate intake (*p* = 0.01 and *p* = 0.01, respectively), but their difference in values were not clinically meaningful. The proportions of patients using lipid-lowering agents significantly increased with increments in carbohydrate intake though there were no significant differences in the use of insulin, oral hypoglycemic agents, and antihypertensive agents.

According to baseline characteristics of daily nutritional intake by the JDCS participants (Table 2), energy intake was significantly lower with increases in the first to third tertiles of the proportion of carbohydrate intake (<50.9%: 1910.3 kcal, 51.0%–56.4%: 1706.3 kcal, and ≥56.5%: 1597.2 kcal, *p* <0.01), and the proportions of fat and protein intake were significantly lower in the higher carbohydrate intake groups (*p* < 0.01, and < 0.01, respectively). The intakes of most micronutrients also lessened significantly with increasing carbohydrate intake. Additionally, the participants with high carbohydrate intake had significantly higher intakes of grain, fruits, and sweets/snacks (*p* < 0.01, < 0.01, and = 0.02, respectively) and significantly lower intakes of soybean and soy products, green-yellow vegetables, other vegetables, seaweed, meat and processed meat, fish and processed fish, eggs, milk and dairy products, oil, and alcoholic beverages (*p* < 0.01 for all) (Table 2).

During the 8-year follow-up period, data were available for 76.5% of the original 1516 participants. Among those in the first to third tertiles of proportions of carbohydrate intake there were 23, 29, and 29 incidents of overt nephropathy, 88, 100, and 89 of diabetic retinopathy, and 40, 38, and 51 of CVD, respectively (Table 3). The crude incidence rates per 1000 patient-years for overt nephropathy, diabetic retinopathy, and CVD were 8.8, 43.2, and 13.8, respectively. There were no notable differences in baseline characteristics between patients who completed the 8-year follow-up and those who did not [11].

Table 3 shows HRs for diabetes complications in relation to the proportion of carbohydrate intake estimated by Cox regression models unadjusted and adjusted for various risk factors. In confounder-adjusted Cox regression, when HRs for complications in patients with carbohydrate intake in the second and third tertiles compared with those with carbohydrate intake in the first tertile were analyzed, no significant differences between proportions of carbohydrate intake and pathogenesis of diabetes complications were found under the fully adjusted model (overt nephropathy: 1.05 (95% CI, 0.54–2.06) and 0.98 (0.40–2.44); diabetic retinopathy: 1.30 (0.90–1.88) and 1.30 (0.78–2.15); and CVD: 0.95 (0.55–1.63) and 1.37 (0.69–2.72)).

**Figure 1:**
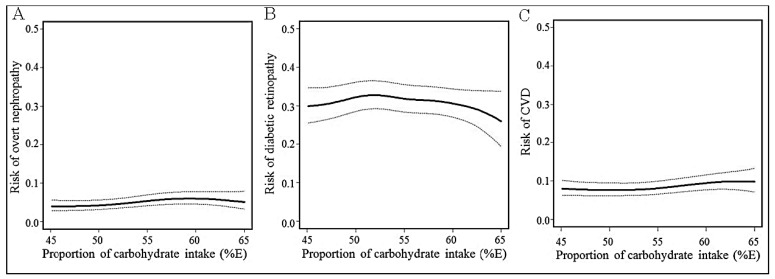
Incidence rate (solid curve) and 95% CI (broken curve) of 8-year overt nephropathy (Panel **A**), diabetic retinopathy (Panel **B**), and CVD (Panel **C**) in relation to proportion of carbohydrate intake at baseline estimated by the generalized additive model. Abbreviation: CVD: cardiovascular disease.

To explore potentially nonlinear relationships between the proportion of carbohydrate intake and the incidence of diabetes complications, we fitted the energy-adjusted generalized additive models (Figure 1). As shown graphically, trends according to the higher or lower proportions of carbohydrate intake were not clearly shown for overt nephropathy, diabetic retinopathy, or CVD.

## 4. Discussion

The optimal proportion of dietary carbohydrate intake recommended as medical nutritional therapy for patients with diabetes has been a topic of ongoing discussion among recent guidelines for diabetes care [3,5]. There have been no large-scale long-term prospective studies that explored the relationship between carbohydrate intake and diabetes complications in patients with diabetes. This 8-year follow-up study of Japanese patients with type 2 diabetes revealed no significant differences between the proportion of carbohydrate intake at baseline (second and third carbohydrate intake tertiles vs. first tertile (referent)) and pathogenesis of diabetes complications, including overt nephropathy, diabetic retinopathy, and CVD. Trends for the incidence of diabetes complications according to the proportion of carbohydrate intake were also not shown when potentially nonlinear relationships were explored by a spline function. The results of this longitudinal study showed that the strict control of carbohydrate intake within a specific range would not be essential to reduce the incidence of diabetes complications among type 2 diabetes patients in Japan.

There are several plausible biochemical and physiological explanations for why diabetes complications develop regardless of different proportions of carbohydrate intake. The main causes of the development of diabetes complications are formation of advanced glycation end products and progression of atherosclerosis. Advanced glycation end products and progression of atherosclerosis can be caused by a complex combination of pathological pathways such as hyperglycemia itself, increased insulin resistance, inflammatory responses, and decreased fluidity of the cell membrane [15]. Insulin resistance in the entire body, including skeletal muscle, liver, and vascular endothelial cells, was reported to be produced by acute and chronic elevations of plasma glucose and free fatty acid levels [16,17]. Therefore, the risks for diabetes complications are elevated when proportions of macronutrition intake are weighted toward carbohydrate or fat. It would also be important to conduct research focused on the details of each macronutrient such as according to the glycemic index, energy density, and type of fatty acid.

According to our current analysis, energy intake was significantly lower in groups with higher proportions of carbohydrate intake, and participants with carbohydrate consumption in the third tertile had about 310 kcal/day lower energy intake compared with those with carbohydrate consumption in the first tertile (first tertile: 1910.3 kcal/day and third tertile: 1597.2 kcal/day). The proportions of fat and protein intake and micronutrient intake also decreased with increasing proportions of carbohydrate intake, which is supported by previous studies [18]. As for intake of food groups, participants with high carbohydrate intake had significantly higher consumption of grain, fruits, and sweets/snacks and lower consumption of the other food groups, with the exception of potato/aroid. These results might indicate that among patients with type 2 diabetes different proportions of carbohydrate intake could be a reflection of differences in intake of various food groups.

To the best of our knowledge, this is the first study on the relationship between the proportion of carbohydrate intake and the incidence of diabetes complications in which patients with type 2 diabetes were prospectively registered based on their HbA1c levels and not retrospectively selected based on self-reported diabetes status. Other strengths include treatment and follow-up plans that were conducted in institutes specializing in diabetes care and adjudication of cardiovascular events by an independent central committee.

Limitations of this study must be considered. First, regarding the potential for bias, the current study did not include patients with type 2 diabetes who had very good glycemic control (HbA1c < 6.5%). Measurement errors in dietary assessments, confounding factors, and informative censoring also cannot be ruled out entirely. We observed significant differences in age, sex, treatment by lipid-lowering agents, and dietary intake across carbohydrate intake groups (Table 1 and Table 2). In our analysis, these confounders were adjusted using Cox regression, but the estimated effects of carbohydrate intake still can be biased because of residual confounding or unmeasured confounders. With regard to informative censoring, we found no notable differences in baseline characteristics between patients who completed the 8-year follow-up and the other patients [11]. Second, as an observational study rather than a randomized trial, we could not conclude cause–effect relationships as to whether medical nutritional treatment managing carbohydrate intake would reduce incident diabetes complications in clinical practice. Another limitation is the accuracy of diabetic retinopathy staging based on clinical diagnosis compared with staging based on seven-field stereo fundus photography [19]. Finally, our results may not be generally applicable to populations with different lifestyles or genetic factors. For example, our study did not include the very well controlled patients whose HbA1c values were less than 6.5%. Additionally, the JDCS patients consumed a “high-carbohydrate, low-fat” diet compared with USA and European patients with type 2 diabetes [20]. In addition, BMI and body weight are markedly different between patients in Japan and USA and European countries [20,21], and Asian patients have a much lower risk of CVD compared with Western patients and a higher risk of end-stage renal disease [22]. The contribution of ethnicity and cultural differences to such differences in patients’ characteristics remains uncertain. Considering ethnic-specific characteristics and large inter-cultural differences is important in exploring effective medical nutritional therapy and further research across other ethnic groups is needed.

## 5. Conclusions

In conclusion, we found that the proportion of carbohydrate intake itself, which would be a reflection of differences in intake of various food groups, was not significantly associated with the incidence of type 2 diabetes complications. The ideal carbohydrate proportions for prevention of each complication could not be specified from the data available. These results suggest that specific proportions of intake of carbohydrate might not be essential for medical nutritional therapy for patients with type 2 diabetes.

## Figures and Tables

**Table 1:** Baseline clinical characteristics of the 1516 patients with type 2 diabetes according to proportion of carbohydrate intake.

			First Tertile		Second Tertile		Third Tertile		
	Total		<50.9%		51.0%–56.4%		≥56.5%		
	(*n* = 1516)		(*n* = 496)		(*n* = 518)		(*n* = 502)		Trend
	Mean	±SD	Mean	±SD	Mean	±SD	Mean	±SD	*p*
Carbohydrate (% energy)	53.6	±6.6	46.4	±4.0	53.6	±1.6	60.7	±3.4	<0.01
Age (year)	58.7	±6.9	57.8	±7.0	58.7	±7.0	59.6	±6.5	<0.01
Women (%)	46.8%		39.5%		51.0%		49.6%		<0.01
Years after diagnosis (year)	11.0	±7.1	11.1	±7.2	11.0	±7.2	10.7	±6.9	0.55
HbA1c (% in NGSP value)	8.3	±1.3	8.2	±1.2	8.4	±1.4	8.3	±1.4	0.01
HbA1c (mmol/mol)	67.3	±14.5	66.0	±13.2	68.5	±15.2	67.3	±14.9	0.01
Fasting blood glucose (mg/dL)	160.7	±43.6	160.0	±39.6	162.1	±46.0	159.9	±44.7	0.62
Diabetic retinopathy (%)	22.2%		22.0%		22.6%		22.1%		0.85
BMI (kg/m^2^)	22.9	±3.0	23.1	±2.9	22.8	±3.0	22.9	±3.0	0.19
Waist circumference (cm)	79.4	±9.0	80.3	±8.8	78.8	±9.4	79.2	±8.7	0.01
SBP (mmHg)	131.4	±16.0	132.2	±16.3	131.3	±15.9	130.8	±15.8	0.20
LDL-cholesterol (mg/dL)	122.4	±32.5	121.0	±35.0	123.1	±30.3	123.1	±32.0	0.24
HDL-cholesterol (mg/dL)	54.5	±17.0	54.8	±17.0	55.6	±17.5	53.1	±16.3	0.86
Triglycerides ^a^ (mg/dL)	102.0	±71.0	101.0	±73.0	98.0	±69.0	104.0	±71.0	0.94
Urine ACR ^a^ (mg/gCre)	16.9	±30.3	16.8	±29.1	17.5	±31.8	16.5	±30.3	0.76
eGFR (mL/min/1.73 m^2^)	87.1	±30.1	87.3	±28.6	87.2	±30.6	86.9	±31.0	0.92
Current smoker (%)	28.7%		30.9%		26.4%		28.8%		0.17
Physical activity (kJ/day) ^a^	589.4	±1097.8	575	±1148	630	±1167	582	±967	0.53
Treated by OHA without insulin (%)	65.8%		64.5%		68.1%		64.9%		0.53
Treated by insulin (%)	20.0%		17.9%		20.7%		21.4%		0.17
Treated by antihypertensive agents (%)	26.4%		27.5%		26.6%		25.2%		0.57
Treated by lipid-lowering agents (%)	24.0%		19.8%		26.1%		25.9%		<0.01

^a^ Median ± interquartile range. Abbreviations: ACR: albumin-to-creatinine ratio; BMI: body mass index; eGFR: estimated glomerular filtration rate; HDL: high-density lipoprotein; LDL: low-density lipoprotein; NGSP: National Glycohemoglobin Standardization Program; OHA: oral hypoglycemic agent; SBP: systolic blood pressure; SD: standard deviation.

**Table 2:** Food groups and nutritional intake per day by the 1516 patients with type 2 diabetes according to proportion of carbohydrate intake at baseline.

			First Tertile		Second Tertile		Third Tertile		
	Total		<50.9%		51.0%–56.4%		≥56.5%		
	(*n* = 1516)		(*n* = 496)		(*n* = 518)		(*n* = 502)		Trend
	Mean	±SD	Mean	±SD	Mean	±SD	Mean	±SD	*p*
Carbohydrate (% energy)	53.6	±6.6	46.4	±4.0	53.6	±1.6	60.7	±3.4	<0.01
**Nutritional intake**									
Energy (kcal/day)	1736.9	±411.9	1910.3	±436.7	1706.2	±371.0	1597.2	±363.6	<0.01
Protein (%energy/day)	15.7	±2.4	17.0	±2.6	15.8	±1.9	14.3	±1.8	<0.01
Fat (%energy/day)	27.6	±5.0	31.7	±4.6	27.8	±3.1	23.4	±3.2	<0.01
Fiber, total (g/day)	14.7	±5.3	15.2	±5.9	14.5	±4.8	14.4	±5.2	0.01
Retinol equivalent (g/day)	1320.2	±532.8	1420.6	±572.2	1308.1	±472.4	1233.6	±535.7	<0.01
Vitamin B1 (g/day)	0.9	±0.3	1.1	±0.3	0.9	±0.2	0.8	±0.2	<0.01
Vitamin B2 (g/day)	1.1	±0.3	1.3	±0.4	1.1	±0.3	0.9	±0.3	<0.01
Vitamin C (g/day)	134.3	±60.7	136.7	±65.3	131.8	±53.6	134.4	±62.8	0.25
Vitamin D (g/day)	11.5	±6.7	14.6	±8.0	11.4	±5.9	8.6	±4.5	<0.01
Sodium (g/day)	4.2	±1.5	4.5	±1.7	4.2	±1.4	3.9	±1.5	<0.01
Calcium (g/day)	639.0	±229.7	721.6	±262.5	645.9	±202.7	550.3	±185.4	<0.01
Iron (g/day)	8.1	±2.6	9.1	±3.0	8.0	±2.2	7.3	±2.1	<0.01
**Intake of food groups**									
Grain (g/day)	191.4	±53.1	174.0	±43.4	190.1	±47.0	210.0	±61.1	<0.01
Potato/Aroid (g/day)	53.5	±45.2	55.6	±51.0	53.8	±41.1	51.1	±43.0	0.25
Soybeans/Soy products (g/day)	71.3	±51.5	90.5	±63.6	69.7	±45.5	54.1	±34.8	<0.01
Fruits (g/day)	133.2	±105.1	120.4	±100.0	128.7	±93.6	150.5	±118.2	<0.01
Green-yellow vegetables (g/day)	138.0	±67.7	146.3	±71.1	136.5	±61.2	131.4	±70.0	<0.01
Other vegetables (g/day)	186.1	±101.9	197.9	±107.0	184.2	±92.9	176.3	±104.4	<0.01
Seaweed (g/day)	2.0	±1.6	2.3	±1.9	2.0	±1.4	1.8	±1.4	<0.01
Meat/Processed meat (g/day)	49.6	±38.3	75.9	±46.5	44.8	±26.1	28.5	±21.3	<0.01
Fish/Processed fish (g/day)	100.1	±60.3	129.6	±71.8	97.6	±50.0	73.7	±42.0	<0.01
Eggs (g/day)	29.0	±16.8	33.1	±16.9	29.6	±16.9	24.3	±15.4	<0.01
Milk/Dairy products (g/day)	170.4	±102.5	189.6	±110.0	180.2	±104.2	141.4	±85.6	<0.01
Sweets/Snacks (g/day)	17.8	±20.5	16.0	±19.3	18.8	±21.8	18.5	±20.3	0.02
Oil (g/day)	16.9	±8.8	21.1	±9.9	16.6	±7.6	12.9	±6.6	<0.01
Alcoholic beverages (g/day)	89.3	±162.2	163.7	±224.0	71.9	±120.0	33.7	±78.8	<0.01

**Table 3:** Cox regression of diabetes complications after 8 years follow up according to proportion of carbohydrate intake at baseline.

	First Tertile		Second Tertile			Third Tertile		
	<50.9%		51.0%–56.4%			≥56.5%		
	HR	95% CI	HR	95% CI	*p*	HR	95% CI	*p*
**Carbohydrate intake at baseline (% Energy)**	52.5	±1.4	57.3	±1.5		63.4	±2.9	
**Overt nephropathy** (*n* = 1275)								
Events/Patients	23/411		29/439			29/425		
Not adjusted	ref		1.21	(0.70 to 2.10 )	0.50	1.23	(0.71 to 2.12 )	0.46
Adjusted ^a^	ref		1.13	(0.63 to 2.02 )	0.68	1.31	(0.72 to 2.37 )	0.38
Adjusted ^b^	ref		1.05	(0.54 to 2.06 )	0.89	0.98	(0.40 to 2.44 )	0.97
Adjusted ^c^	ref		1.19	(0.66 to 2.15 )	0.56	1.32	(0.71 to 2.44 )	0.37
**Diabetic retinopathy** (*n* = 936)								
Events/Patients	88/305		100/321			89/310		
Not adjusted	ref		1.13	(0.86 to 1.50 )	0.41	1.00	(0.74 to 1.34 )	0.99
Adjusted ^a^	ref		1.12	(0.82 to 1.51 )	0.48	1.00	(0.72 to 1.38 )	1.00
Adjusted ^b^	ref		1.30	(0.90 to 1.88 )	0.17	1.30	(0.78 to 2.15 )	0.31
Adjusted ^c^	ref		1.14	(0.84 to 1.55 )	0.41	1.06	(0.76 to 1.48 )	0.73
**CVD** (*n* = 1353)								
Events/Patients	40/443		38/458			51/452		
Not adjusted	ref		0.90	(0.57 to 1.40 )	0.63	1.29	(0.85 to 1.95 )	0.23
Adjusted ^a^	ref		0.90	(0.57 to 1.44 )	0.67	1.24	(0.79 to 1.96 )	0.35
Adjusted ^b^	ref		0.95	(0.55 to 1.63 )	0.84	1.37	(0.69 to 2.72 )	0.36
Adjusted ^c^	ref		0.88	(0.55 to 1.41 )	0.58	1.21	(0.76 to 1.93 )	0.42

^a^ Adjusted for age, sex, BMI, HbA1c, diabetes duration, systolic blood pressure, LDL-cholesterol, HDL-cholesterol, triglycerides, treatment by insulin, treatment by antihypertensive agents, treatment by lipid-lowering agents, current smoker, alcohol intake, energy intake, and physical activity; ^b^ Further adjusted for intakes of saturated fatty acids, monounsaturated fatty acids, polyunsaturated fatty acids, omega 6 fatty acids, omega 3 fatty acids, cholesterol, dietary fiber, and sodium.; ^c^ Adjusted for age, sex, BMI, HbA1c, diabetes duration, systolic blood pressure, LDL-cholesterol, HDL-cholesterol, triglycerides, treatment by insulin, treatment by antihypertensive agents, treatment by lipid-lowering agents, current smoker, alcohol intake, energy intake, and physical activity and excluding patients whose energy intake to basal metabolic rate ratio was less than 0.9. Abbreviation: CVD: cardiovascular disease; CI: confidence interval.
